# Exploring types of focused factories in hospital care: a multiple case study

**DOI:** 10.1186/1472-6963-10-154

**Published:** 2010-06-07

**Authors:** Eelco Bredenhoff, Wineke AM van Lent, Wim H van Harten

**Affiliations:** 1 School of Management and Governance, University of Twente, The Netherlands; 2 Netherlands Cancer Institute - Antoni van Leeuwenhoek Hospital, The Netherlands

## Abstract

**Background:**

Focusing on specific treatments or diseases is proposed as a way to increase the efficiency of hospital care. The definition of "focus" or "focused factory", however, lacks clarity. Examples in health care literature relate to very different organizations.

Our aim was to explore the application of the focused factory concept in hospital care, including an indication of its performance, resulting in a conceptual framework that can be helpful in further identifying different types of focused factories. Thus contributing to the understanding of the diversity of examples found in the literature.

**Methods:**

We conducted a cross-case comparison of four multiple-case studies into hospital care. To cover a broad array of focus, different specialty fields were selected. Each study investigated the organizational context, the degree of focus, and the operational performance. Focus was measured using an instrument translated from industry. Data were collected using both qualitative and quantitative methods and included site visits. A descriptive analysis was performed at the case study and cross-case studies level.

**Results:**

The operational performance per specialty field varied considerably, even when cases showed comparable degrees of focus. Cross-case comparison showed three focus domains. The product domain considered specialty based focused factories that treated patients for a single-specialty, but did not pursue a specific strategy nor adapted work-designs or layouts. The process domain considered delivery based focused factories that treated multiple groups of patients and often pursued strategies to improve efficiency and timeliness and adapted work-designs and physical layouts to minimize delays. The product-process domain considered procedure based focused factories that treated a single well-defined group of patients offering one type of treatment. The strategic focusing decisions and the design of the care delivery system appeared especially important for delivery and procedure based focused factories.

**Conclusions:**

Focus in hospital care relates to limitations on the patient group treated and the range of services offered. Based on these two dimensions, we identified three types of focused factories: specialty based, delivery based, and procedure based. Focus could lead to better operational performance, but only when clear strategic focusing decisions are made.

## Background

Hospital care is under pressure to increase quality and decrease cost [1]. As a result, hospitals look into the opportunities offered by concepts from business. One of these concepts is the so-called focused factory concept. Implementing "focused factories", aimed at specific treatments or diseases, is proposed as a way to increase the efficiency of hospital care [2,3].

The focused factory was introduced in manufacturing as: *"a plant established to focus the entire manufacturing system on a limited, concise, manageable set of products, technologies, volumes, and markets precisely defined by the company's strategy, its technology, and its economics" *[4,5]. Focusing aims to prevent that distinct products are produced in one and the same manufacturing system, as this will lead to trade-offs that hinder the fulfillment of product requirements and deteriorate the competitiveness of the organization [4-8]. In services, focus requires organizations to segment their markets and develop focused delivery systems for each segment [9,10]. The objective of this segmentation is to identify relatively homogeneous groups of customers. Often, this reveals smaller, more predictable, manageable patterns in demand [9].

Both in manufacturing and services, different types of focus are identified. The literature describes focus on products (product lines or markets), processes (internal capabilities), and competitive priorities (sometimes described as "order winners") [6,11-13]. We summarized the characteristics used to differentiate between different foci in Table 1. These characteristics show two focus dimensions: one relates to variety in product or customer requirements, the other to variety in the processes or technologies used in the delivery system.

**Table 1:** Characteristics in literature to differentiate between types of focused factories

Product characteristics	Process characteristics
Product variety [6,11]: The number and volumes of products or parts produced in the manufacturing system (product life cycle: evolving from low-volume one of a kind products towards high volume standardized products) Customer intimacy [10]: The degree to which the customer interacts with the service, combined with the degree to which the service is customized for the consumer Customer influence [40]: The degree to which the customer influences the service by his presence, participation or interaction with the system Customization [40,41]: The degree of customization (discretion) allowed for in the service delivery system Uniqueness of services [42]: The degree of discretion, freedom, and decision making power in selecting their service combined with the degree of repeatability of the service Volume of customers [41]: The number of customers processed per business unit per period	Process variety [6,11]: The number of technologies used in the manufacturing system (process life cycle: evolving from job-shop flexible systems towards continuous standardized systems) Labor intensity [10]: The ratio of labor costs incurred to the value of the plant and equipment Number of routes in the delivery system [42] The number of unique pathways (routes) customers can take as they move through the service system during delivery of the service.

The literature on focus in hospital care, however, describes focus as a diffuse mix of treatment characteristics, patient characteristics, specialty characteristics, and organizational aspects [2,14-16]. Examples of focused factories consider very different types of organizations, such as cancer clinics [2], trauma centers [15], specialty hospitals [3,14,17], and ambulatory surgery centers [14,16,18]. The definition of "focus" or "focused factory" in hospital care lacks clarity. This is problematic in two ways.

First, the diversity of examples and diffuse mix of characteristics shows that the focused factory concept in hospital care is not well understood. Probably the closest thing to a definition of focus in hospital care is offered by Herzlinger, who describes focused factories as (multidisciplinary) organizations based on common objectives (e.g. the treatment of specific patient groups) [2]. Herzlinger, in effect, calls for a change from professional-centered to patient-centered (and process-centered) organizations. This is in contrast with the traditional view of hospital organizations as professional bureaucracies, characterized by extensive division of labor and organizational units that are based on specialties [19,20]. However, creating these (multidisciplinary) organizational units, might solve some of the problems associated with the traditional hospital organization, such as; coordination problems, a work-around culture, lack of team-work, and high numbers of handovers [21-23]. Although Herzlinger appears to suggest that these focused organizations should be independent, Schneider [17] argues there is no reason why hospitals could not create these kinds of organizational units themselves. Requirements on the organizational context, e.g. the operations strategy and design of the care delivery system, remain unclear.

Second, evaluating the efficiency of focused factories becomes difficult when there is so much diversity. Conclusions on the operational performance of focused factories are mixed. Several case studies on specialty hospitals and centers for ambulatory surgery report improved efficiencies, higher patient satisfaction, comparable or decreased mortality rates and less adverse outcomes on the hospital level [16,18,24,25]. Other studies found higher re-admission rates for more complex patients [26,27], or reported incidents of specialty hospitals calling in emergency care from a general hospital [28,29]. A recent cost comparison of physician-owned specialty hospitals and full-service providers in 3 US states showed that orthopedic and surgical specialty hospitals had significantly higher levels of cost inefficiency [30]. In the econometric literature a large number of hospital efficiency studies are described (see [31] for an overview). Most of these studies show inefficiencies, but offer little theoretical explanation for the efficiency differences. A recent study into the efficiency of hospitals and their departments in the Netherlands [32], suggests these efficiency differences result from the way hospitals are organized.

To understand the diversity in examples of focused factories in hospital care, further studies are required. Similar to manufacturing and services, hospital care delivery systems are influenced by varieties. For example: varieties in; case-mix; the experience and capacities of medical doctors, specialty groups, and other (nursing) staff; and the availability of infrastructure and medical technologies [19,33,34]. In response to these varieties, it seems likely that, in hospital care, different types of focused factories may exist. These might differentiate depending on the degree of customization, the variation in services needed, and possible other characteristics that relate to varieties in hospital care provision.

This paper aims to explore the application of the focused factory concept in hospital care, including indications of its performance, resulting in a conceptual framework that can be helpful in further identifying different types of focused factories. We performed multiple case studies in four specialty fields, investigating the degrees of focus, the organizational context, and the operational performance. We used the two dimensions of focus from business literature to group cases with similar degrees of focus. Thus we hope to contribute to the understanding of the characteristics of the care delivery system and operations strategy for different types of focus.

## Methods

### Study design and selection

The study consisted of a cross-case comparison of four, separately performed, multiple-case studies in different specialty fields. Within each specialty field, we performed a comparative multiple case study with embedded units of analysis [35]. This means that within each single case (e.g. a hospital) attention was given to a subunit or subunits (the actual focused factories).

The fields for the case studies were selected to correspond with- and reflect the variety of focus examples in the literature [2,3,14,17]. In order to cover a broad array of focus in hospital care, we used the characteristics differentiating between different foci in the literature (see Table 1) as guidelines. There were obvious differences in the volumes, variety in case-mix, and procedures offered between the studied fields of: medical oncology, orthopedics and total knee implants, cataract care, and low-complex elective surgery.

Using the characteristics and identified fields, we sampled conveniently, primarily selecting hospitals in the Netherlands, but included international good practice cases on medical oncology and cataract care due to the limited number of cases in the Netherlands. We aimed for at least two hospitals and three units of analysis per specialty field. An overview of the cases and units of analysis included is presented in Table 2.

**Table 2:** Overview of the included cases and units of analysis, per specialty field

Medical Oncology							
Case	Unit	Region				Treatment places	

1	1	EU				30	
2	2	EU				13	
3	3	US				24	
	4	US				29	
	5	US				48	
	6	US				7	
							
**Orthopedics and Total Knee Implants**							

Case	Unit	Region			Inpatient beds		

4	7	NL			42		
	9	NL			6		
5	8	NL			78		
	10	NL					
							
**Cataract Care**							

Case	Unit	Region					No. of annual cataract surgeries

6	11	NL					2630
7	12	UK					6309
8	13	US					7366
							
**Low-complex Elective surgery**							

Case	Unit	Region	Operating rooms	Day care beds	Inpatient beds		

9	14	NL	2	18	-		
10	15	NL	4	24	32		
11	16	NL	4	26	16		
12	17	NL	2		37		
	18	NL	2	10	-		

### Measures

We investigated the organizational context by looking at the operations strategy (related to focusing), including the implications for the design of the care delivery system. This design, the organizational structure, is believed to influence organizational outcomes [36-38]. We studied the use of standardized procedures, the use of dedicated (physical) layouts [38], the applied planning routines, and the team composition [37].

The degree of focus was investigated using a measurement instrument translated from industry. In the literature, only two attempts to measure degrees of focus were found.

Mukherjee [7] used a quantitative approach, calculating focus scores for one plant, based on volume and variety of products and parts. Pesch and Schroeder [39] used a mixed approach, calculating degree of focus scores for multiple plants, based on a questionnaire investigating variety in products, volumes and competitive priorities. Since the approach of Pesch and Schroeder [39] had been tested in multiple organizations, we chose to adapt their measurement instrument to the specialty fields investigated. Using the customization, the uniqueness of services, and the number of identifiable processes in the delivery system (see table 1), we adapted the instrument to measure two axes of focus. One axis measured the product focus, investigating the volume and variety in patients treated. The other axis measured the process focus, investigating the volume and variety in specialties involved and services offered. The measurement instrument is supplied in additional file 1.

The operational performance was investigated per specialty field, by looking at process indicators; such as utilization, lead times, and the costs of the resources used. The indicators chosen varied per specialty field, depending on the availability of data.

### Data collection

Data were collected using both qualitative and quantitative methods. Quantitative data were retrieved from annual reports and, when necessary, provided by the organization. Qualitative data were collected through interviews and observations during site visits. Each study was performed by the authors, or as a master thesis project closely supervised by the authors. Case study protocols per specialty field were used.

### Analysis

First, we discussed the similarities and differences between the individual (sub) units' organizational context, degrees of focus, and operational performance per specialty field. We analyzed whether the organizational context and degrees of focus could explain differences in operational performance.

Next, we grouped the (sub) units of all four multiple case studies into a framework, based on the degrees of product and process focus. In this cross-case comparison we discussed similarities (and differences) in the organizational context between units in the same group. Replication logic [35] was used to define types of focused factories in hospital care.

## Results

### Medical oncology: chemotherapy day units in the EU and US

We studied the chemotherapy day units (CDU) of three comprehensive cancer centers in the EU and US. An earlier pilot study, comparing complete cancer centers, lead us to conclude this lower level of analysis was required to explain differences. In total, 6 CDUs were studied. The results (see Table 3) show that all centers applied dedicated layouts and standardized procedures for treatments. Planning routines, however, differed, and were based on: the total daily workload, the availability of a bed and nurse, and the arrival time and treatment duration. Only one case had an explicit strategic objective to maximize the utilization of beds and nurses, which was influenced by limited floor capacity and budget constraints.

**Table 3:** Medical Oncology

Unit	1	2	3	4	5	6
**Organizational context**						
*Focusing decisions/operations strategy*	Focus on patient centeredness and access/waiting times	Maximizing utilization/access times	Focus on patients' safety and prevention of claims/long opening hours to allow patients to come after work/short access times.			
*Standardized procedures*	Yes	Yes	Yes	Yes	Yes	Yes
*Dedicated lay-out*	Yes, only suitable for medication related treatments	Yes, only suitable for medication related treatments	Yes, only suitable for medication related treatments	Yes, special air ventilation etc for bone marrow patients	Yes, only suitable for medication related treatments	Yes, only suitable for medication related treatments
*Planning routine:*						
1 Occupancy times of beds	No, calculated workload is based on this information	Yes	Yes			
2 Insight into available beds at a certain moment	No	Yes	No			
3 Workload	Yes, total work-load of the day is calculated, not workload at a specific time	Manually checked by head nurse.	Done manually			
4 Planning is visualized	No	Yes	Yes			
5 Relative importance of experience for the planning	High	Moderate/low	High			
*Team composition*	Stable	Rotational shift with other department(s)	Stable			
						
**Degrees of focus**						
*Product focus*	85%	85%	78%	85%	85%	100%
*Process focus*	78%	78%	83%	83%	83%	83%
						
**Operational performance**						
Indexed average number of patients treated per bed per month (not corrected for differences in opening hours)	44	100	77			
Indexed average number of patient visits per month per total CDU staff	58	100	44			

Indexed = the best performing CDU received a score of 100, the other CDU received a relative score compared to the best performing CDU. Table partly based on [43]

Although all studied cases scored similar degrees of focus, the operational performance differed considerably. Both the number of patient visits per bed and the number of visits per employee showed differences up to 56%. Although we were unable to correct in detail for differences in opening hours and nurse staffing between cases, we are confident considerable differences will remain when corrected. These were unlikely to be explained by the minor differences in focus. Planning procedures and staff scheduling rather seemed to explain the variation in operational performance. Higher scores of focus did not correspond with higher performance. The fit between the strategic choices on focus on patient or product categories and the design of the service delivery system appears to cause different operational performance.

### Orthopedics care and total knee implants: comparing an orthopedics center and a general hospital in the Netherlands

We studied the orthopedics departments and (joint care-) total knee implants groups of a general hospital (GH) and an orthopedics center (OC) in the Netherlands. The results (see Table 4) show that only the general hospital pursued efficiency improvements as a strategic objective, and developed a joint-care program (unit 8) directed at total hip and total knee implants. Work-designs and the layout were adapted, for instance to create a 'living room' that enabled group wise treatment and rehabilitation. Planning routines differed per medical doctor and, remarkably, joint-care patients were not always operated sequentially. The orthopedics center performed all activities on one location. It had not developed any special programs as a strategic choice, and left initiatives in this regard to its medical doctors. Planning routines used fixed times for surgeries. Various procedures were, however, operated upon in a random sequence, suggesting that minimizing changeover times was not an explicit objective. Both cases showed frequent changes in team-composition.

**Table 4:** Orthopedics care and total knee implants

Unit	7	8	9	10
**Organizational context**				
*Focusing decisions/operations strategy*	No clear strategy	Strategy pursuing efficiency for total knee implants	No clear strategy	No clear strategy for total knee implants
*Standardized procedures*	No	Yes	No	No
*Dedicated lay-out*	Layout was not dedicated Diagnostics, preoperative screening and surgery took place on different locations	Layout adapted to create 'living room' that enabled group wise treatment and rehabilitation of knee implants patients Diagnostics, preoperative screening and surgery took place on different locations	Layout was not (really) dedicated Diagnostics, preoperative screening and surgery took place on one location	Layout was not (really) dedicated Diagnostics, preoperative screening and surgery took place on one location
*Planning routine*	Different planning routines per MD	Different routines per MD. Joint-care patients not always operated sequentially	Standardized planning routines, using fixed surgical times Sequence of surgeries was 'random' ignoring negative changeover effects	Standardized planning routines, using fixed surgical times Sequence of surgeries was 'random' ignoring negative changeover effects
*Team composition*	Frequent changes in team composition	Frequent changes in team composition	Frequent changes in team composition	Frequent changes in team composition
				
**Degrees of focus**				
*Product focus*	47%	75%	47%	56%
*Process focus*	56%	75%	56%	56%
				
**Operational performance**				
Average duration of surgery (min)	48	110	90	90
Preparation time for knee surgery (min)		30		40
Average Length of stay	5,6	5,0	5,9	6,9
Utilization of ward	78%		88%	
Overhead cost per discharged patient (€)	107		290	

The degrees of focus indicate we encountered two types of focused factories. One focused on the treatment of orthopedics patients in general, and one on the treatment of patients for knee implants. Although the degrees of focus for knee implants groups were higher, some efficiency parameters scored lower (GH: utilization of wards, OC: length of stay) and the comparison of overhead costs favored the general hospital. Only the knee implant patients treated in the joint-care program of the general hospital showed shorter lengths of stay. The differences in the average duration of orthopedics surgery and the length of stay for orthopedics patients are probably caused by the lower complexity of the case-mix of the general hospital. The higher volume of knee surgeries in the orthopedics center probably explains why their average duration of knee surgery is shorter. The limited number of cases does not allow firm conclusions. We could thus not establish a unidirectional relation between focus and efficiency.

### Cataract care: cataract clinics at eye hospitals in the Netherlands, United Kingdom and United States

We studied the cataract care of three eye hospitals, located in the Netherlands (11), the United Kingdom (12), and United States (13). The results (see Table 5) show that two cases aimed at efficient patient flows and short waiting times. Both cases created a dedicated cataract clinic to realize efficient care delivery and adapted their work-designs to offer diagnostics and preoperative assessments on the same day. These cases showed extensive division of labor allocating specific tasks to nursing staff, while in the other case medical doctors performed most tasks. This last case seemed especially geared towards preventing liability and, as a consequence, performed redundant preoperative assessments and -reviews.

**Table 5:** Cataract care

Unit	11	12	13
**Organizational context**			
*Focusing decisions/operations strategy*	Strategy pursuing efficiency	Strategy to reduce the no. of visits in order to realize lead time ≤ 18 weeks	Strategy pursuing open access, prevent medical liability
*Standardized procedures*	Yes	Yes	Yes
*Dedicated lay-out*	Cataract clinic with dedicated day-surgery operating rooms	Cataract clinic with dedicated day-surgery operating rooms	Clinic applied general operating rooms
*Planning routine*	One stop diagnostics and scheduling of surgery Preoperative assessments according to open access model	One stop diagnosis, preoperative assessment and scheduling of surgery	One stop diagnosis, preoperative assessment and scheduling of surgery for clinic patients Most diagnostics (91% of the patients) take place outside the hospital at the practice of affiliated ophthalmologists
*Team composition*	Extensive division of labor allocating tasks to nurses	Extensive division of labor allocating tasks to nurses	MDs perform most tasks
			
**Degrees of focus**			
*Product focus*	94%	94%	94%
*Process focus*	84%	84%	84%
			
**Operational performance**			
Visits/patient	3.2	3.0	3.6
Lead time (days)	91.0	109.2	15.0
- Access time to outpatient clinic	25.0	31.5	0.0
- Waiting time for surgery	66.0	77.7	15.0

The organizational outcomes for unit 13 exclude diagnostics (as these take place outside the hospital). If these outside diagnostics were taken into account, the degree of focus score on process focus could deteriorate to 78% or 72%

Although all cases had similar degrees of focus, the highest efficiency seemed to be found in the UK (12), followed by NL (11) and lastly the US (13). As is supported by the results on the number of visits per treatment episode and the use of day-surgery operating rooms over more expensive general operating rooms. The operational performance seemed most influenced by the different operational strategies pursued, aiming for: efficiency (11), timeliness (12), and medical liability (13). These strategies reflected the characteristics of the national reimbursements systems.

### Low-complex elective surgery: hospital-owned centers for low-complex elective surgery in the Netherlands

We studied five hospital-owned centers for low-complex elective surgery in four teaching hospitals in the Netherlands. We investigated the perceived advantages of their care delivery systems. The results (see Table 6) show that most hospitals applied strategies aimed at improving efficiency and timeliness of care. This resulted in the (re)development of smaller hospitals as centers for elective surgery offering low-complex surgery in day-care, short-stay, or both. All cases performed one-stop shop preoperative assessments, and only admitted patients with low-risk physical conditions. Physical layouts were adapted to reduce transportation times and delays. Planning routines differed; sometimes a separate planning department made the schedule, sometimes the medical specialty itself. In most cases, staff worked both in the elective surgery center and the general operating room.

**Table 6:** Low-complex elective surgery

Unit	14	15	16	17	18
**Organizational context**					
*Focusing decisions/operations strategy*	No clear strategy The day-surgery clinic evolved as result of a hospital wide cost reduction program	Strategy to improve efficiency and timeliness of elective surgery All low-complex elective surgery was concentrated in one center for elective surgery	Strategy to improve efficiency and timeliness of elective surgery A center for elective surgery in day care and short stay was developed	Strategy to improve efficiency and timeliness of day-surgery	Strategy to improve efficiency and timeliness of elective surgery
*Standardized procedures*	Protocols for most treatments and standardized discharge letters	Protocols for most treatments	Protocols for most treatments	Protocols for most treatments	Protocols for most treatments
*Dedicated lay-out*	OR's and ward located on the same floor to reduce transportation times. OR applied a holding and recovery to minimize delays	OR's and wards located in the same building on separate floors. Dedicated transportation elevators were used to reduce transportation times and delays	OR's and wards located on the same floor to reduce transportation times. OR applied a holding and recovery to minimize delays	OR's and ward located on the same floor to reduce transportation times. OR applied a combined holding/recovery to minimize delays	The day-surgery clinic integrated the OR's, ward, holding and recovery into one unit. Patients 'walk' to the OR, reducing transportation times
*Planning routine*	Preoperative assessments on appointment Surgical planning made by planning specialty	Preoperative assessments on appointment Surgical planning made by planning department or MDs (depending on specialty)	Preoperative assessments on appointment and open access Surgical planning made by planning department	Preoperative assessments on appointment Surgical planning made by planning specialty	Preoperative assessments on appointment Surgical planning made by planning specialty
*Team composition*	Frequent changes in team composition	Frequent changes in team composition	Frequent changes in team composition	Fixed team compositions	Fixed team compositions
**Degrees of focus**					
*Product focus*	44%	44%	44%	44%	44%
*Process focus*	72%	66%	66%	66%	75%
					
**Operational performance**					
*Average duration of surgery (min)*					
- General surgery	54	56	24	-	58
- ENT surgery	11	37	18	59	21
- Orthopedics surgery	32	52	21	46	47
- Plastic surgery	-	68	26	57	21
					
*HHI per specialty (hospital total)*					
- General surgery	0.068 (0.016)	0.093 (0.024)	0.170 (0.024)	-	0.148 (0.025)
- ENT surgery	0.222 (0.057)	0.096 (0.093)	0.154 (0.057)	0.337 (0.014)	0.109 (0.014)
- Orthopedic surg.	0.433 (0.092)	0.097 (0.065)	0.211 (0.027)	0.261 (0.072)	0.362 (0.072)
- Plastic surgery	-	0.047 (0.324)	0.067 (0.083)	0.155 (0.091)	0.985 (0.091)

The Herfindahl-Hirschmann Index (HHI) was used to calculate the concentration of the surgical procedures offered per specialty per unit (see organizational outcomes). Low variety corresponds with 1, high variety with 0.

The degrees of product focus were similar for all cases, while two had higher scores on process focus, probably related to reduced variety, as they only performed elective surgery in day-care. All centers for elective surgery treated a less-complex case mix (compared to the general hospital) and performed surgical procedures with shorter average duration and lower variation in duration. The Herfindahl-Hirschmann Index (HHI), calculated based on the surgical procedure codes and their volumes, showed reductions in variety by 2 - 4 times compared to the hospital, depending on the specialty. In one case (16) a medical doctor even reported a shorter average duration for one type of surgical procedure, compared to the same procedure performed on the general OR (thought to be caused by the experience of staff). Data were, however, insufficient to compare the exact utilization of the operating rooms, as each case used different definitions to collect data. Although obtaining comparable data proved difficult, observations showed that strategic decisions on the introduction of focus actually led to more efficient processes (14, 16 and 18). We observed short turnover times on the OR and wards. Furthermore, all studied centers used less expensive OR resources compared to a general hospital, as only a limited set of surgical procedures was performed.

### Cross-case analysis: towards a framework of focused factories

The results of the four specialty fields did not show a clear relationship between the degrees of focus and the organizational context. Operational performance seemed to depend on focus, strategic choices, and the related organizational context. Units that combined a high degree of focus with clear strategic objectives aimed at efficiency or timeliness, often, showed higher degrees of efficiency or timeliness. When focus scores were similar, variations in efficiency seemed to be related to differences in operating procedures or operations strategy.

Combining high degrees of focus with a well-defined operations strategy and work-designs, thus, appeared more important in realizing higher efficiencies than the degree of focus alone. It is of importance, to gain insights in the organizational characteristics of different types of focused factories.

**Figure 1:**
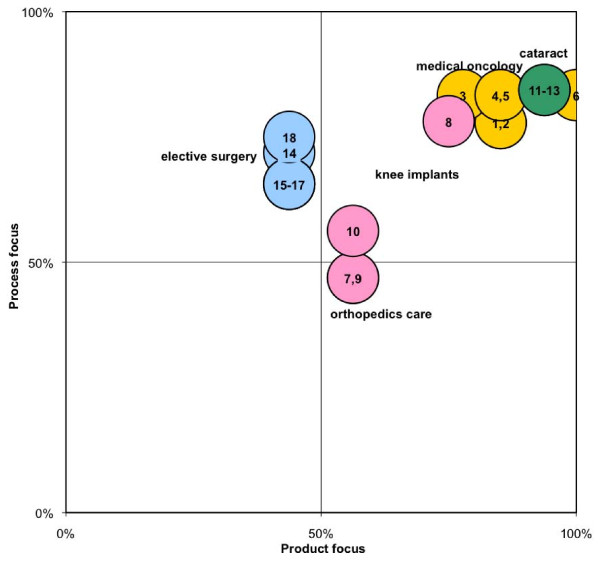
**Position of the units in the focus matrix**. Based on the degrees of focus on product and process, the positions of the units are depicted in the focus matrix. Four specialty field were studied. Medical oncology (yellow) considered two EU units (1, 2) and four US cases (4 - 6). Orthopedics care and knee implants considered the orthopedic departments (7, 9) and knee implants (8, 10) of a general hospital and orthopedics center in the Netherlands. Cataract care (green) considered one NL (11), one UK (12), and one US (13) case. Elective surgery (blue) considered five centers for low-complex elective surgery in the Netherlands (14 - 18).

We positioned the 18 (sub)units of the four specialty fields into a focus matrix, based on their degrees of focus on product and process (see Figure 1). Units in the field of orthopedics care and knee implants showed high degrees of focus on products, with the units for knee implants also showing higher degrees of focus on processes. Units in the field of elective surgery showed high degrees of focus on the process axis, but lower degrees of focus on products. The units in the fields of medical oncology and cataract care showed high degrees of focus on both products and process axes.

This focus matrix can be used as a framework for identifying different types of focused factories. We propose three domains of focus, related to high scores on the separate axis: the product domain, the process domain, and the product-process domain. For each domain we compared the similarities (dissimilarities) in the organizational context of all units in the same domain (see Table 7).

**Table 7:** Cross case comparison, per focus domain of the focus matrix

Focus Domain	Product domain	Process domain	Product-Process domain
**Degrees of focus**			
*Product focus*	50-100	0-50	50-100
*Process focus*	0-50	50-100	50-100
			
**Units**	7,9	14,15,16,17,18	1,2,3,4,5,6,8,10,11,12,13
			
**Organizational context**			
*Operations strategy (focusing decisions)*	No clear operations strategy	Strategy aimed at high efficiency and short lead times	Strategy aimed at efficiency and/or timeliness
*Standardized procedures*	No	Yes	Yes
*Dedicated lay-out*	Activities grouped in one location	Layouts adapted to minimize distances and delays	Layouts adapted to enable more efficient ways of treating patients (e.g. group rehabilitation). Distances are minimized
*Planning routines*	Planning routines differed per MD	Most units showed one stop shop arrangements for diagnostics and preoperative assessments Planning routines were both centralized and decentralized	More or less standardized
*Team composition*	Frequent changes in team composition	Most units showed frequent changes in team composition. Two units showed stable team compositions	Frequent changes in team composition

#### The product domain: specialty based focused factories

Both orthopedic departments are found in the product domain. These organizations implemented a high degree of focus by limiting the types of patients treated, related to their specialty. Neither pursued a specific operations strategy. Work-designs and physical layouts of both cases were not adapted and served more or less a general purpose. As these organizations treat a single-specialty, they can be defined as specialty based focused factories.

#### The process domain: delivery based focused factories

All centers for low-complex elective surgery are found in the process domain. These organizations aimed at efficient delivery of specific types of treatments: low-complex, low-risk elective surgical procedures offered by multiple specialties. The operations strategies aimed for high efficiencies and short lead times. Processes were standardized and the physical layouts adapted to minimize distances. The care delivery systems were characterized by standardization and predictability. Two highly focused cases worked with stable team-compositions. As these organizations treat patients based on delivery characteristics, they can be defined as delivery based focused factories.

#### The product-process domain: procedure based focused factories

The chemotherapy day units, centers for cataract care, and the knee implants (join-care) units are found in the product-process domain. These organizations treated a single, specific, group of patients and offered one single (type of) treatment or a single surgical procedure. The majority of the cases pursued strategies aimed at efficiency or timeliness, and consequentially adapted work-designs and the physical layouts. Work-processes were standardized, describing well-defined tasks. Physical layouts enabled more efficient ways of treating patients (such as group rehabilitation) or minimized physical distances. Planning routines differed, but were more or less standardized. Surprisingly, most organizations changed team-compositions frequently. As these organizations treat a single group of patients for a single (type of) treatment, they can be defined as procedure based focused factories.

## Discussion

Although the majority of units we studied were procedure based focused factories, we believe that the proposed distinction between different types of focus leads to a better understanding of the relations between focus, operational choices, and the operational performance of an organization. The fit between strategic focusing decisions and the design of the care delivery system appears especially important for delivery and procedure based focused factories.

### Strengths and limitations of this study, suggestions for further research

To our knowledge, our analysis, including the development of a measurement instrument, was the first attempt to measure the degree of hospital focus in a structured way. Our approach makes it possible to study and compare types and degrees of focus in different specialty fields.

The focus measurement instrument has some drawbacks. Differences between the fields and national healthcare systems make it necessary to adapt the measurement instrument to each specialty field, for instance through defining the ICD code limits. Thus the validity and consistency of the instrument needs further study, especially as we could only include 18 cases. Looking at the difference in focus scores, further studies into the scale and cut-off points that distinguish between different domains of focused factories are needed.

The structured case analysis, provided insights in the relations between the degrees of focus, the design of care delivery systems, and the operational performance. We concluded that the applied operations strategy and resulting adaptations to the care delivery system within focused factories explain the differences in performance. This fit between strategic focusing decisions and care delivery design, the operations strategy, appears vital. Although the degree of focus seemed less important in predicting higher efficiencies or timeliness, a higher degree of focus indicates reduced variety. This variety reduction might offer organizations the opportunity to develop a well-focused operations strategy. Further studies into the role of the operations strategy in focused factories are required. Developing a measure of the degree of fit that can be added to the scoring system, seems worthwhile.

The proposed framework provides insights in the main characteristics of three types of focused factories. It offers a way for identifying similar types of focused factories, based on product and process foci. We cannot exclude the possibility that not all types of focused factories in hospital care are covered. For instance, diagnostic departments, which might require different focus- and operational choices, were not included in our research. Further studies in other fields are therefore required to validate the framework.

A recent study of Schneider et al [17], covering 70 cases, describes factors that are associated with the economic success of specialty hospitals, such as clinical efficiency and procedural economies of scale. They conclude that there is as yet no conclusive material supporting either the advantages or disadvantages of this type of hospital and suggest that the same type of benefits might be attainable for units within larger hospitals. Schneider et al underline the need for a theory or conceptual framework to identify areas of specialization that would lead to benefits for consumers and payers. This aligns very well with our plea for further research into types and benefits of focus factories in hospital care.

## Conclusions

Our study shows that focus in hospital care relates to limitations on the patient group treated and the services or treatments offered. Four multiple case studies in the fields of medical oncology, orthopedics, cataract care, and elective surgery showed different scores of focus on product and process.

Process focus appeared more often to be related to strategic choices considering the organizational structure and the design of the care delivery system. Product focus appeared to be related to limiting the patient groups treated, having only implicit consequences for the organizational structure. Based on the differences in these degrees of focus, we distinguish three main types of focused factories: specialty based, delivery based and procedure based focused factories.

The results suggest that focus can lead to higher productivity and utilization, but only when clear strategic focusing decisions are made. The applied strategic choices and their consequences for the design of the care delivery system seem more important in explaining differences in operational performance than focus scores as such. This might be one of the reasons why studies into the effects of focused factories, including the econometric literature, show such mixed results.

## Competing interests

No conflicting interests can be indicated. WvH is member of the executive board of one of the involved chemotherapy day units studied in the field of medical oncology.

## Authors' contributions

EB co-supervised three case studies, performed one case study, (re)wrote the first draft of the paper, and revised the text. WvL performed one of the case studies and contributed to the text. WvH supervised three case-studies and contributed to- and revised the text. All authors read and approved the final manuscript.

## Pre-publication history

The pre-publication history for this paper can be accessed here:

http://www.biomedcentral.com/1472-6963/10/154/prepub

## Supplementary Material

Additional file 1 **Measurement instrument focused factories in hospital care**. This file presents the measurement instrument used by the researchers to assess the degrees of focus of focused factories in hospital care. Two dimensions are investigated: 1) the degree of product focus, by investigating the volume and variety in patients treated, 1) the degree of process focus, by investigating the volume and variety in specialties involved and services offered. The file can be viewed in Word. Click here for file
