# Supplementary material for: Exploring types of focused factories in hospital care: a multiple case study
Source: BMC Health Serv Res. 2010 Jun 7;10:154. doi: 10.1186/1472-6963-10-154 (PMC2904334; doi:10.1186/1472-6963-10-154)
Supplement: Additional file 1 — Measurement instrument focused factories in hospital care. This file presents the measurement instrument used by the researchers to assess the degrees of focus of focused factories in hospital care. Two dimensions are investigated: 1) the degree of product focus, by investigating the volume and variety in patients treated, 1) the degree of process focus, by investigating the volume and variety in specialties involved and services offered. The file can be viewed in Word. [file 1472-6963-10-154-S1.DOC]

# Measurement instrument focused factories in hospital care

## Measuring the degree of focus on patients (patient variety)

These questions gather information about: the groups of patients that are treated, the volume of these groups, and the variety (between and within these groups). These questions give us information about the customer needs and particularities.

### Question 1

We would like to know how many patient groups are treated in your organization. A patient group will be considered as all patients that belong to one chapter of the ICD classification (please see Figure 1).

| Chapter | Blocks | Title |
| --- | --- | --- |
| I | **A00-B99** | Certain infectious and parasitic diseases |
| II | **C00-D48** | Neoplasms |
| III | **D50-D89** | Diseases of the blood and blood-forming organs and certain disorders involving the immune mechanism |
| IV | **E00-E90** | Endocrine, nutritional and metabolic diseases |
| V | **F00-F99** | Mental and behavioural disorders |
| VI | **G00-G99** | Diseases of the nervous system |
| VII | **H00-H59** | Diseases of the eye and adnexa |
| VIII | **H60-H95** | Diseases of the ear and mastoid process |
| IX | **I00-I99** | Diseases of the circulatory system |
| X | **J00-J99** | Diseases of the respiratory system |
| XI | **K00-K93** | Diseases of the digestive system |
| XII | **L00-L99** | Diseases of the skin and subcutaneous tissue |
| XIII | **M00-M99** | Diseases of the musculoskeletal system and connective tissue |
| XIV | **N00-N99** | Diseases of the genitourinary system |
| XV | **O00-O99** | Pregnancy, childbirth and the puerperium |
| XVI | **P00-P96** | Certain conditions originating in the perinatal period |
| XVII | **Q00-Q99** | Congenital malformations, deformations and chromosomal abnormalities |
| XVIII | **R00-R99** | Symptoms, signs and abnormal clinical and laboratory findings, not elsewhere classified |
| XIX | **S00-T98** | Injury, poisoning and certain other consequences of external causes |
| XX | **V01-Y98** | External causes of morbidity and mortality |
| XXI | **Z00-Z99** | Factors influencing health status and contact with health services |
| XXII | **U00-U99** | Codes for special purposes |

Figure 1: Main topics ICD classification, needed to answer question I, II and III

How many of the ICD classification chapters describe your patients? Please consider the period of a year (preferably 2005). Only one answer possible.

1. one or two chapters (4)
2. three chapters (2)
3. more than three chapters (0)

### Question 2

We are interested in the variation in patients (defined by ICD code) treated. Each chapter of the ICD classification consists of a number of blocks. What percentage of blocks in the chapters indicated in question 1, describes your patients? Please consider the period of a year (preferably 2005). If multiple chapters were indicated in question 1, describe the overall percentage. Only one answer possible.

1. 0-20% (4)
2. 20-40% (3)
3. 40-60% (2)
4. 60-80% (1)
5. >80% (0)

### Question 3

We are interested in how patients are distributed between the blocks. Large differences between blocks suggest high variety in patient needs. Please indicate how you value the variety in the number of patients between blocks of a chapter.

Please indicate how the variety in numbers of patients between blocks is perceived

1. very high (0)
2. high (1)
3. moderate (2)
4. low (3)
5. very low (4)

### Question 4

The health of a patient can be described by the Classification of the American Society of Anesthesiologists (ASA). Each ASA class (Please see Figure 2) describes a different physiological situation of a patient. Patients classified as ASA 1 have fundamentally different needs than patients classified as ASA 4. Limiting an organization to serve patients classified to only one or a few ASA classes suggests a higher degree of focus on patient groups.

| Class | Description |
| --- | --- |
| I | The patient is normal and healthy |
| II | The patient has mild systemic disease that does not limit their activities (e.g., controlled hypertension or controlled diabetes without systemic sequellae) |
| III | The patient has moderate or severe systemic disease, which does limit their activities (e.g., stable angina or diabetes with systemic sequellae). |
| IV | The patient has severe systemic disease that is a constant potential threat to life (e.g., severe congestive heart failure, end-stage renal failure). |
| V | The patient is morbid and is at substantial risk of death within 24 hours, with or without surgery. |

Figure 2: ASA classification, needed to answer question 4

How many ASA classes describe the patients receiving the care of your organization?

1. one or two ASA classes (4)
2. three ASA classes (2)
3. more than three ASA classes (0)

## Measuring the degree of focus on processes (service variety)

These questions gather information about the number of involved specialties, the number of offered services, the variety in services and some patient characteristics that strongly influence the predictability of care delivery system.

### Question 5

We are interested in the number of specialties that are involved in the delivery of care in your organization. Radiology and Anaesthesiology must be excluded in this number.

How many specialties are involved in your organization?

1. one specialty (4)
2. two specialties (2)
3. more than 3 specialties (0)

### Question 6

We are interested in the number of subspecialties within the specialties in your organization. Please indicate which option applies.

How many subspecialties are involved in your organization?

1. one subspecialty (4)
2. two subspecialties (2)
3. more than 3 subspecialties (0)

Question 7 Does your organization has its own (dedicated) radiology department? Please, indicate if your organization does not use X-ray, CT or MRI imaging in serving its patient groups.

1. Yes, we have our own radiology department (4)
2. No, but the radiology department is ours for
   the majority (90%) of the day (3)
3. No, but (part of) the radiology department
   is ours on specific (parts of) days (2)
4. No, but we have a higher priority at the
   radiology department (1)
5. No, the radiology department is a shared
   resource out of our control (0)
6. No, we don’t use X-ray, CT or MRI in serving
   patients (not applicable) (4)

### Question 8

The number of services your organization offers is measured by the number of treatments or diagnostics offered. We identify medical, surgical, and radiation treatments. We therefore distinguish between four types of services: 1) medical treatments, 2) surgical treatments, 3) radiation treatments, and 3) diagnostics. Consultation is seen as an integral part of care delivery, and is therefore not seen as a distinct service.

Please indicate how many of these types of services your organization offers

1. Only one of these types of services (4)
2. Two of these types of services (2)
3. More than two types of services (0)

### Question 9

For each type of service offered, we are interested in the variety within this service. In the case of medical treatments, we are interested in the number of medicines used, and the number methods for administering these medicines. In the case of surgical treatments we are interested in the number of different surgical procedures performed. In the case of radiation treatments we are interested in the variety within treatment times. In the case of diagnostics we are interested in the number of diagnostic services (techniques/ technologies) used such as lab-tests, radiology, physical exam, ECG, etc.

Please indicate, for each service your organization offers, which options applies. The different options are modeled on examples in literature and expert opinion.

***medical***

1. few medicines, few methods (4)
2. multiple medicines, few methods (3)
3. moderate medicines and methods (2)
4. Few medicines multiple methods (1)
5. Multiple medicines and methods (0)

***surgical***

1. < 50 different procedures (4)
2. 50-100 different procedures (3)
3. 100-250 different procedures (2)
4. 250-500 different procedures (1)
5. > 500 different procedures (0)

***radiation***

1. very high variety in treatment times (0)
2. high variety in treatment times (1)
3. moderate variety in treatment time (2)
4. low variety in treatment times (3)
5. very low variety in treatment times (4)

***diagnostics***

1. one or two diagnostics used (4)
2. three or four diagnostics used (2)
3. five or more diagnostics used (0)

If multiple services were indicated, use the lowest score when establishing the degree of focus on offered services.

### Question 10

The ASA classes (see Figure 2), used to describe the health of patients, can seriously influence the predictability of the delivery of care. Although the ASA classification depends on patients, we also see it as an indicator for interruptions in the delivery of care. Patients in the high ASA classes require a care delivery system that is able to rapidly respond to unpredictable events. Patients in the low ASA classes mainly require routine care. The ASA classes that occur in the care delivery system therefore indicate the predictability of this system.

Please, indicate which option applies for your organization.

1. no patient has an ASA classification higher than 2 (4)
2. Less than 50% of the patients have an ASA classification equal to 3,
   no patients have an ASA classification higher than 3 (3)
3. more than 50% of the patients have an ASA classification larger or equal to 3,
   but no patients have an ASA classification equal or higher than 4 (2)
4. more than 25% of the patients have an ASA classification higher or equal to 4 (1)
5. more than 50 % of the patients have an ASA classification higher or equal to 4 (0)

### Question 11

Another process characteristic is the percentage of inpatient and outpatient admissions compared to the total number of admissions.

Please indicate which option applies for your organization

1. more than 95% of all patients is treated either inpatient or outpatient (4)
2. less than 95% of all patients is treated outpatient, but inpatient LOS < 5 (2)
3. less than 95% of all patients is treated either inpatient or outpatient (0)

### Question 12

Also the number of urgent cases tells us something about the services offered, and their predictability. It is possible to focus on non-urgent cases, as it is to focus on urgent cases.

Please indicate which option applies for your organization.

1. more than 95% or less than 5 % of all patients are
   urgent cases (4)
2. other (0)

## Calculate the degree of focus and classify the type of focus factory

We calculate the percentages of the maximum degree focus score that was attained. This gives us a score of 0 – 100 percent on both dimensions.

**Focus on patients**

Sum the scores of the questions 1 to 4. Divide this score by the maximum possible score of 16 points. Multiply the result with 100. This leads to a degree of focus score of 0 – 100.

**Focus on services**

Sum the scores of the questions 5 to 11. Divide this score by the maximum possible score of 28 points. Multiply the result with 100. This leads to a degree of focus score of 0 – 100.
